# Moderating role of live microbe between chronic inflammatory airway disease and depressive symptoms

**DOI:** 10.3389/fnut.2025.1572178

**Published:** 2025-04-28

**Authors:** Wenqiang Li, Qian He, Jingshan Bai, Youli Wen, Zefu Hu, Zhiping Deng, Qian Huang

**Affiliations:** ^1^Department of Pulmonary and Critical Care Medicine, Zigong First People's Hospital, Zigong, Sichuan, China; ^2^Department of Obstetrics and Gynecology, West China Second University Hospital, Sichuan University, Key Laboratory of Birth Defects and Related Diseases of Women and Children (Sichuan University) of Ministry of Education, Chengdu, Sichuan, China; ^3^Department of Respiratory Medicine, Xiong'an Xuanwu Hospital, Xiong'an, Hebei, China; ^4^Department of Pulmonary and Critical Care Medicine, Dazhou Dachuan District People's Hospital (Dazhou Third People's Hospital), Dazhou, Sichuan, China

**Keywords:** chronic inflammatory airway disease, depressive symptoms, dietary live microbe, cross-sectional studies, microbiotherapy

## Abstract

**Purpose:**

Our study aims to investigate the impact of dietary live microbe on the relationship between chronic inflammatory airway diseases (CIAD) and depressive symptoms.

**Methods:**

We selected data from the NHANES database from 2007 to 2020. First, we explored the relationship between CIAD and depressive symptoms using logistic regression analysis. And subgroup analyses were conducted to demonstrate the relationship and whether there was an interaction effect between the two in each subgroup. Then, we further analyzed the effect of live microbe on depressive symptoms in CIAD patients. And subgroup analyses were conducted to assess whether the effect of dietary viable microbial levels on depressive symptoms held true in each subgroup and whether there was an interaction effect.

**Results:**

A study included 23,072 participants, of whom 5,111 were diagnosed with CIAD, and 5,110 had live microbial information available. Multivariate logistic regression analysis revealed that, compared to those without CIAD, individuals with CIAD had an increased risk of depressive symptoms. Subgroup analysis indicated that, except for educational level and smoking status, all other subgroups demonstrated that CIAD increased the risk of depressive symptoms. Additionally, within the CIAD population, a higher level of live microbe was associated with a reduced risk of depressive symptoms. It is implied that live microbe can negatively modulate the relationship between CIAD and depressive symptoms. Subgroup analysis further showed no significant interaction effects across subgroups (*p* > 0.05).

**Conclusion:**

Chronic inflammatory airway diseases can increase the risk of developing depressive symptoms. Dietary live microbe negatively modulate the relationship between CIAD and depressive symptoms. High levels of dietary live microbe significantly reduced the risk of depressive symptoms in patients with CIAD.

## 1 Introduction

Chronic inflammatory airway disease (CIAD) is a chronic disease in which inflammation involves the upper and/or lower airways, characterized by airway inflammation, airway obstruction and airway remodeling, with bronchial asthma and chronic obstructive pulmonary disease (COPD) being the most common (1, 2). In recent years, with environmental changes and socio-economic development, epidemiology shows that the number of patients with CIAD is increasing (3–5).

Mental illness is one of the major diseases contributing to the increased global burden of disease (6). Depression is a mental illness that affects mood, behavior, and overall health. A recent study showed a global prevalence of depression of 290 million, an increase of approximately 60% from 1990 (7). In recent years, as research has progressed, inflammation has been confirmed as a critical disease modulator, promoting susceptibility to depression (8). Both diabetes and coronary heart disease have been shown to have a close association with the occurrence of depression (9, 10). Some researchers have even developed prediction models for depression symptoms in patients with diabetes using nationwide large sample data (11). However, for chronic inflammatory respiratory diseases, there is a lack of research evidence to confirm their potential relationship with depressive symptoms.

Metabolites of intestinal microbe can reach the lungs through the blood circulation and lymphatic system, participating in regulating immune responses and inflammatory processes in the lungs (12). Animal experiments demonstrate that transplantation of fecal microbe from healthy animals ameliorates emphysema and alveolar destruction in mice exposed to cigarette smoke (13). Moreover, in mouse models with limited antibody repertoires, gut transplants of probiotics can improve allergic responses by reducing airway eosinophil infiltration, thereby lowering the incidence of acute asthma attacks (14). Increasing evidence suggests that the mechanisms by which gut microbes ameliorate lung disease are related to immune regulation (15) at the same time, the relationship between gut microbes and brain neurotransmitters should not be overlooked, and there are implications for the development of depression symptoms (16) whereas the relationship between dietary viable microbes and the modulation of depressive symptoms in CIAD is unclear.

Therefore, we used the National Health and Nutrition Examination Survey (NHANES) database to analyze the relationship between CIAD and depressive symptoms. We also explored whether there is a moderating effect of dietary live microbe on depressive symptoms in patients with CIAD. We aim to uncover the potential impact of live microbes in this complex biopsychosocial syndrome, thereby providing new perspectives and scientific basis for intervention strategies. Through this research, we intend to deepen our understanding of these diseases and explore potential therapeutic and preventive measures to alleviate patient suffering.

## 2 Materials and methods

### 2.1 Data sources

The NHANES database^1^ is a publicly accessible database with rich content and reliable data. It is a national health and nutrition survey program in the United States that began in 1999. The survey reaches various levels of the population, employing a complex multistage, stratified, cluster sampling method to select nationally representative participants. And it is conducted every 2 years, with about 5,000 people surveyed each time. NHANES staff collects data through household interviews, questionnaires, and tests. The program was approved by the National Center for Health Statistics (NCHS) Ethics Review Society and all participants signed an informed consent form.

### 2.2 Participants

**Figure 1:**
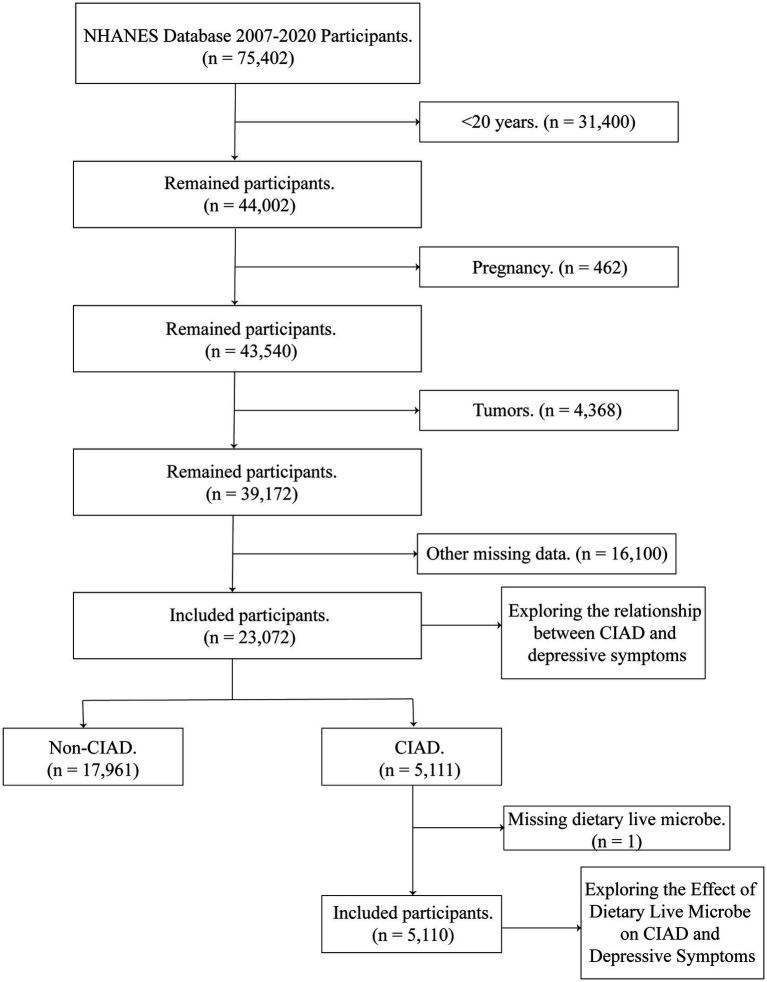
Participant screening flowchart. CIAD, chronic inflammatory airway diseases.

We selected NHANES database 2007–2020 participants (*n* = 75,402) for 7 cycles. The following conditions were excluded: (1) <20 years of age (*n* = 31,400); (2) pregnancy (*n* = 462); (3) tumor (*n* = 4,368); and (4) other missing items (*n* = 16,100). Ultimately, we included a total of 23,072 for analysis, of which 5,111 were CIAD patients. The effect of live microbe on depressive symptoms was further explored in the remaining 5,110 CIAD patients after removing the missing live microbe (Figure 1).

### 2.3 The definition of CIAD

The CIAD in our study was determined primarily by questionnaires from the NHANES database, including asthma, chronic bronchitis, emphysema, and chronic obstructive pulmonary disease. Participants were asked whether they had been told by a doctor or other health professional that they had asthma, chronic bronchitis, emphysema, or COPD. Participants who answered “yes” were recognized as having these diseases.

### 2.4 Depressive symptoms

The diagnosis of depressive symptoms was determined primarily on the basis of Patient Health Questionnaire 9 (PHQ-9) scores. The PHQ-9 scores range from 0 to 3 for each item and 0–27 for the total score. A PHQ-9 score greater than or equal to 10 is diagnosed as depressive symptoms and is widely used in the study of depressive symptoms (17, 18).

### 2.5 Assessment of the levels of live microbe in the diet

The levels of live microbe in the NHANES database were determined by four experts (MLM, MES, RH, and CH) in the field based on dietary categories (a total of 48 subgroups, containing 9,388 food codes). Participants’ dietary intake was collected by NHANES staff through a face-to-face interview format. Participants were asked about their diet the previous day (midnight-midnight), including all foods and beverages. Different foods contained different amounts of live microbe. The levels of live microbe contained in the food was inferred from previous relevant studies, authoritative reviews or food processing methods and categorized as low (Lo, <10_4_ CFU/g), medium (Med, 10_4_–10_7_ CFU/g) and high (Hi, >10_7_ CFU/g) (19–21). “Lo” refers to pasteurized foods, such as milk, prepared meats, gravies, etc. “Med” refers to unpeeled fresh fruits and vegetables. “Hi” refers to unpasteurized fermented foods and probiotic supplements.

### 2.6 Research variables

We included a number of variables that may have had an impact on this study. Demographic variables included age (20–44, 45–64, ≥65), sex (Male, Female), marriage (Married/Living with Partner, Widowed/Divorced/Separated or Never married), and race (White, Black, Mexican, or Other). Variables reflecting socioeconomic status included education level (Below high school diploma, High school diploma, Above high school diploma), poverty-to-income ratio (PIR) (<1.3, 1.3–3.5, >3.5), and household insurance (Yes, No). The PIR ranged from 0 (no family income) to 5 (family income at least five times the annual federal poverty level). Health and behavioral variables included smoking (Never smoked, Former smoker, Current smoker), drinking (Yes, No), and body mass index (BMI) (<25, ≥25 and <30, ≥30 kg/m^2^). Comorbidities included diabetes mellitus (DM) (Prediabetes, Yes, No), hypertension (Yes, No), and cardiovascular disease (CVD) (Yes, No). Never smoker, former smoker, and current smoker were defined as having smoked less than 100 cigarettes in their lifetime, more than 100 in their lifetime but not currently smoking, more than 100 cigarettes in their lifetime and still smoking, respectively (22).

### 2.7 Statistical analysis

We included data from 7 cycles of the NHANES database and selected appropriate weights for analysis according to database usage requirements. All statistical analyses were performed using R software version 4.3.2 and *p* < 0.05 (two-sided) indicated statistical significance. The variables in our study were converted to categorical variables, expressed as percentages, and compared between groups using the chi-square test. Logistic regression analysis was used to assess the odds ratio (OR) of CIAD with the occurrence of depressive symptoms, and 95% confidence intervals (CI) were calculated.

We constructed four models for analysis. The crude model did not adjust for any factors. Model 1 adjusted for age, sex, race, and education level. Model 2 adjusted for PIR, household insurance, smoking, and drinking based on model 1. Model 3 adjusted for BMI, DM, hypertension, and CVD based on model 2. And subgroup analyses by age, sex, race, smoking, BMI, and education level demonstrated the relationship and whether there was an interaction effect between the two in each subgroup. Subsequently, we further analyzed the impact of live microbe on depressive symptoms in patients with CIAD. We also conducted subgroup analyses by age, sex, race, smoking, BMI, and educational level to evaluate whether the effect of dietary live microbe on depressive symptoms is consistent across these subgroups and to assess any potential interaction effects.

## 3 Results

### 3.1 Baseline characteristics of all participants

A total of 23,072 participants were included in this study, of which 2,061 suffered from depressive symptoms and 5,111 suffered from CIAD. As can be observed in Table 1, among the depressive symptom population, a higher percentage of the participants were female, young to middle-aged, Caucasian, Married/Living with Partner, PIR < 1.3, obese, and had a above high school diploma, could afford to purchase household insurance, and were current smokers or had a drinking habit. In all groups, there were significant differences between those with and without depressive symptoms (all *p* < 0.05). A higher proportion of those with depressive symptoms had comorbid DM, hypertension, CVD, or CIAD compared with those without, implying that comorbidities with these conditions may increase the risk of developing depressive symptoms. However, the population distribution of the included participants was not homogeneous among the variables, and whether the above phenomenon is meaningful requires further analysis.

**Table 1:** Baseline characteristics of participants with and without depressive symptoms.

Variable	Total	Non-depressive	Depressive	*P*
Age group (%)				<0.001
20–44	10,579 (49.13)	9,675 (49.26)	904 (47.70)	
45–64	8,153 (36.78)	7,290 (36.34)	863 (41.57)	
≥65	4,340 (14.09)	4,046 (14.40)	294 (10.73)	
Sex (%)				<0.0001
Female	11,592 (50.48)	10,291 (49.40)	1,301 (62.38)	
Male	11,480 (49.52)	10,720 (50.60)	760 (37.62)	
Marriage (%)				<0.0001
Married/Living with Partner	13,672 (61.74)	12,760 (63.21)	912 (45.53)	
Never married	4,603 (20.98)	4,125 (20.58)	478 (25.44)	
Widowed/Divorced/Separated	4,797 (17.28)	4,126 (16.21)	671 (29.04)	
Race (%)				0.02
Black	5,066 (11.32)	4,614 (11.14)	452 (13.28)	
Mexican	3,495 (8.72)	3,202 (8.75)	293 (8.36)	
Other	5,037 (13.67)	4,595 (13.47)	442 (15.88)	
White	9,474 (66.30)	8,600 (66.64)	874 (62.49)	
Education level (%)				<0.0001
High school diploma	5,318 (23.55)	4,813 (23.14)	505 (28.06)	
Below high school diploma	5,179 (14.38)	4,486 (13.54)	693 (23.58)	
Above high school diploma	12,575 (62.07)	11,712 (63.32)	863 (48.36)	
PIR (%)				<0.0001
<1.3	7,384 (22.54)	6,303 (20.69)	1,081 (42.98)	
1.3–3.5	8,642 (34.71)	7,953 (34.67)	689 (35.23)	
>3.5	7,046 (42.74)	6,755 (44.65)	291 (21.79)	
Household insurance (%)				<0.0001
No	5,172 (18.41)	4,622 (17.84)	550 (24.72)	
Yes	17,900 (81.59)	16,389 (82.16)	1,511 (75.28)	
Drinking (%)				0.03
No	3,097 (10.19)	2,861 (10.34)	236 (8.45)	
Yes	19,975 (89.81)	18,150 (89.66)	1825 (91.55)	
Smoking (%)				< 0.0001
Former	5,238 (23.28)	4,803 (23.55)	435 (20.26)	
Never	12,922 (56.23)	12,079 (57.77)	843 (39.30)	
Current	4,912 (20.49)	4,129 (18.68)	783 (40.43)	
BMI (kg/m^2^) (%)				<0.0001
<25	6,517 (29.39)	6,030 (29.71)	487 (25.92)	
25–30	7,427 (32.26)	6,894 (32.86)	533 (25.72)	
≥30	9,128 (38.34)	8,087 (37.43)	1,041 (48.36)	
DM (%)				<0.0001
Prediabetes	506 (1.83)	453 (1.76)	53 (2.59)	
No	19,763 (89.24)	18,146 (89.85)	1,617 (82.47)	
Yes	2,803 (8.93)	2,412 (8.39)	391 (14.95)	
Hypertension (%)				<0.0001
No	15,218 (70.14)	14,082 (71.21)	1,136 (58.44)	
Yes	7,854 (29.86)	6,929 (28.79)	925 (41.56)	
CVD (%)				
No	20,920 (92.66)	19,216 (93.23)	1704 (86.32)	
Yes	2,152 (7.34)	1795 (6.77)	357 (13.68)	
CIAD (%)				<0.0001
No	17,961 (78.25)	16,648 (79.50)	1,313 (64.44)	
Yes	5,111 (21.75)	4,363 (20.50)	748 (35.56)	

PIR, poverty-to-income ratio; BMI, body mass index; DM, diabetes mellitus; CVD, cardiovascular disease; CIAD, chronic inflammatory airway disease.

### 3.2 Association of CIAD with depressive symptoms

To clarify the relationship between CIAD and depressive symptoms, four models were constructed. All four models showed that CIAD was strongly associated with depressive symptoms, and that having CIAD increased the risk of developing depressive symptoms (OR > 1, *p* < 0.0001, Table 2). In the full model, we found that having CIAD increased the risk of developing depressive symptoms by approximately 62% [OR 1.62, 95% CI (1.37, 1.90)], which was statistically significant (*p* < 0.0001, Table 2). Therefore, we need to pay great attention to the physical and mental health of CIAD patients and provide them with appropriate assistance.

**Table 2:** Relationship between CIAD and depressive symptoms.

Variable	Crude model	*P*	Model 1	*P*	Model 2	*P*	Model 3	*P*
	OR (95%CI)		OR (95%CI)		OR (95%CI)		OR (95%CI)	
CIAD								
No	Reference	/	Reference	/	Reference	/	Reference	/
Yes	2.14 (1.84,2.49)	<0.0001	2.01 (1.71,2.35)	<0.0001	1.76 (1.50,2.07)	<0.0001	1.62 (1.37,1.90)	<0.0001

Crude model: no adjustment for any factor.

Model 1: adjusted for age, sex, race, and education level.

Model 2: adjusted for PIR, household insurance, smoking, and drinking based on model 1.

Model 3: adjusted for BMI, DM, hypertension, and CVD based on model 2.

PIR, poverty-to-income ratio; BMI, body mass index; DM, diabetes mellitus; CVD, cardiovascular disease; CIAD, chronic inflammatory airway disease.

To investigate the association between CIAD and depressive symptoms across different population strata and assess potential interaction effects, we conducted subgroup analyses stratified by age, sex, race, smoking, BMI, and education level. The results showed that having CIAD increased the risk of depressive symptoms in all subgroups, but there was an interaction effect in the education level and smoking groups (*p* < 0.05). This suggests that the impact of CIAD on depressive symptoms varied significantly across different educational levels and smoking intensity. The risk of depressive symptoms was most significantly increased by having CIAD among those with below high school diploma and former smokers, which were 2.85 and 2.5 times higher than those who did not have CIAD, respectively [below high school diploma, OR 2.85, 95% CI (2.21, 3.67); Former smokers, OR 2.50, 95% CI (1.83, 3.43), *p* < 0.0001 for both, Table 3]. This suggests that CIAD patients with lower educational level or a history of smoking require heightened vigilance for the development of depressive symptoms, and early psychological assessment should be prioritized in these populations to mitigate adverse outcomes.

**Table 3:** Subgroup analysis on the relationship between CIAD and depressive symptoms.

Variable	Non-depressive	Depressive	*P*	*P* for interaction
Sex				0.2
Female	Reference	1.92 (1.60,2.31)	<0.0001	
Male	Reference	2.37 (1.83,3.07)	<0.0001	
Age group				0.1
20–44	Reference	1.93 (1.57,2.36)	<0.0001	
45–64	Reference	2.21 (1.79,2.72)	<0.0001	
≥65	Reference	3.01 (2.05,4.42)	<0.0001	
Race				0.62
Other	Reference	2.06 (1.53,2.78)	<0.0001	
White	Reference	2.26 (1.81,2.82)	<0.0001	
Mexican	Reference	2.05 (1.39,3.03)	<0.001	
Black	Reference	1.83 (1.45,2.31)	<0.0001	
Smoking				0.01
Never	Reference	1.44 (1.15,1.81)	0.002	
Former	Reference	2.50 (1.83,3.43)	<0.0001	
Current	Reference	2.27 (1.77,2.91)	<0.0001	
BMI (kg/m^2^)				0.41
<25	Reference	2.31 (1.63,3.27)	<0.0001	
25–30	Reference	1.76 (1.33,2.35)	<0.001	
≥30	Reference	2.15 (1.78,2.59)	<0.0001	
Education level				0.02
High school diploma	Reference	2.33 (1.76,3.08)	<0.0001	
Below high school diploma	Reference	2.85 (2.21,3.67)	<0.0001	
Above high school diploma	Reference	1.79 (1.45,2.22)	<0.0001	

BMI, body mass index.

### 3.3 Baseline characteristics of CIAD patients with different levels of dietary live microbe

A total of 5,111 CIAD patients were enrolled in this study, removing one patient who lacked information on dietary live microbe, leaving 5,110. The number of low, medium and high levels of dietary live microbe were 2,063, 1,956 and 1,091, respectively. Table 4 shows the specific baseline conditions. We observed that there were differences between low, medium and high level dietary live microbe in all groups except hypertension and drinking (*p* < 0.05). The proportion of people with hypertension, DM, CVD and obesity was lower in the high level dietary live microbe compared to the low and medium level dietary live microbe.

**Table 4:** Baseline characterization based on different levels of dietary live microbe.

Variable	Total	Lo	Med	Hi	*P*
Age group (%)					0.03
20–44	2,288 (48.37)	967 (51.25)	806 (44.70)	515 (49.67)	
45–64	1845 (37.02)	722 (36.04)	733 (38.89)	390 (35.65)	
≥65	977 (14.61)	374 (12.71)	417 (16.40)	186 (14.68)	
Sex (%)					<0.001
Female	2,787 (55.92)	1,022 (50.37)	1,123 (59.64)	642 (58.41)	
Male	2,323 (44.08)	1,041 (49.63)	833 (40.36)	449 (41.59)	
Marriage (%)					0.03
Married/Living with Partner	2,742 (57.61)	1,030 (53.29)	1,076 (59.76)	636 (60.67)	
Never married	1,115 (22.90)	491 (24.80)	392 (21.77)	232 (21.82)	
Widowed/Divorced/Separated	1,253 (19.49)	542 (21.91)	488 (18.47)	223 (17.50)	
Race (%)					<0.0001
Black	1,260 (12.48)	632 (16.38)	439 (11.69)	189 (8.00)	
Mexican	442 (5.49)	160 (5.61)	198 (5.92)	84 (4.70)	
Other	997 (12.59)	356 (12.38)	405 (13.25)	236 (11.89)	
White	2,411 (69.44)	915 (65.63)	914 (69.14)	582 (75.41)	
Education level (%)					<0.0001
High school diploma	1,197 (23.97)	557 (28.85)	442 (23.00)	198 (18.37)	
Below high school diploma	1,095 (14.74)	531 (19.23)	403 (13.86)	161 (9.55)	
Above high school diploma	2,818 (61.28)	975 (51.92)	1,111 (63.14)	732 (72.07)	
PIR (%)					<0.0001
<1.3	1869 (27.19)	890 (35.07)	652 (23.61)	327 (21.09)	
1.3–3.5	1819 (33.85)	737 (34.45)	717 (34.61)	365 (31.86)	
>3.5	1,422 (38.96)	436 (30.48)	587 (41.78)	399 (47.05)	
Household insurance (%)					0.002
No	951 (16.10)	439 (19.57)	341 (14.56)	171 (13.39)	
Yes	4,159 (83.90)	1,624 (80.43)	1,615 (85.44)	920 (86.61)	
Drinking (%)					0.09
No	525 (8.09)	222 (8.26)	215 (9.05)	88 (6.39)	
Yes	4,585 (91.91)	1841 (91.74)	1741 (90.95)	1,003 (93.61)	
Smoking (%)					<0.0001
Former	1,322 (26.27)	494 (24.16)	538 (28.46)	290 (26.07)	
Never	2,370 (47.36)	854 (40.21)	948 (50.18)	568 (53.52)	
Current	1,418 (26.36)	715 (35.62)	470 (21.36)	233 (20.41)	
BMI (kg/m^2^) (%)					0.04
<25	1,292 (27.36)	503 (25.68)	507 (27.89)	282 (28.99)	
25–30	1,448 (28.92)	562 (26.60)	553 (29.53)	333 (31.39)	
≥30	2,370 (43.72)	998 (47.73)	896 (42.58)	476 (39.62)	
DM (%)					0.02
Prediabetes	129 (2.23)	44 (1.55)	55 (2.79)	30 (2.39)	
No	4,183 (85.95)	1,679 (85.97)	1,575 (84.05)	929 (88.77)	
Yes	798 (11.82)	340 (12.48)	326 (13.16)	132 (8.84)	
Hypertension (%)					0.13
No	3,022 (64.90)	1,203 (62.14)	1,152 (65.93)	667 (67.36)	
Yes	2088 (35.10)	860 (37.86)	804 (34.07)	424 (32.64)	
CVD (%)					
No	4,370 (88.35)	1751 (87.92)	1,658 (87.52)	961 (90.19)	
Yes	740 (11.65)	312 (12.08)	298 (12.48)	130 (9.81)	

Lo, <10^4^ CFU/g; Med, 10 (4)-10^7^ CFU/g; Hi, >10^7^ CFU/g. PIR, poverty-to-income ratio; BMI, body mass index; DM, diabetes mellitus; CVD, cardiovascular disease; CIAD, Chronic Inflammatory Airway Disease.

### 3.4 The role of dietary live microbe in CIAD and depressive symptoms

We explored the effect of dietary live microbe on depressive symptoms in patients with CIAD. In Table 5, all four models we constructed showed that high levels of dietary live microbe reduced the risk of depressive symptoms compared to low levels of dietary live microbe in patients with CIAD. In the full model, compared to individuals with low levels of dietary live microbe, those with high levels of dietary live microbe showed a reduction in the risk of depressive symptoms in CIAD patients by approximately 39% [OR 0.61, 95% CI (0.47, 0.80), *p* < 0.001]. No significant effect was observed among those with medium levels (*p* > 0.05). This suggests that high levels of live microbe in the diet can significantly negatively modulate the relationship between CIAD and depressive symptoms, and can significantly reduce the risk of depressive symptoms in patients with CIAD.

**Table 5:** Association between different levels of dietary live microbe and depressive symptoms in a CIAD population.

Variable	Crude model	*P*	Model 1	*P*	Model 2	*P*	Model 3	*P*
	OR (95%CI)		OR (95%CI)		OR (95%CI)		OR (95%CI)	
Dietary live microbe								
Lo	Reference		Reference		Reference		Reference	
Med	0.62 (0.47, 0.80)	<0.001	0.67 (0.50, 0.88)	0.005	0.79 (0.59, 1.04)	0.09	0.79 (0.59, 1.05)	0.11
Hi	0.45 (0.34, 0.58)	<0.0001	0.52 (0.40, 0.67)	<0.0001	0.61 (0.47, 0.80)	<0.001	0.61 (0.47, 0.80)	<0.001

Crude model: no adjustment for any factor.

Model 1: adjusted for age, sex, race, and education level.

Model 2: adjusted for PIR, household insurance, smoking, and drinking based on model 1.

Model 3: adjusted for BMI, DM, hypertension, and CVD based on model 2.

Lo, <10^4^ CFU/g; Med, 10 (4)-10^7^ CFU/g; Hi, >10^7^ CFU/g. PIR, poverty-to-income ratio; BMI, body mass index; DM, diabetes mellitus; CVD, cardiovascular disease; CIAD, chronic inflammatory airway disease.

To clarify whether this relationship was retained and whether there was an interaction effect in different levels of the population. We further performed subgroup analyses stratified by age, sex, race, smoking, BMI, and education level. In Table 6, the results showed no interaction effect in all subgroups (*p* > 0.05). However, this role of high levels of dietary live microbe to negatively modulate the effects of CIAD on depressive symptoms may have been present only in the subgroups of sex, BMI, above high school diploma, never smoked or current smoker, White, and 20–64 years old (*p* < 0.05). It was especially most significant in the normal BMI group, with a reduction of about 69%.

**Table 6:** Subgroup analysis on the relationship between different levels of dietary live microbe and depressive symptoms in a CIAD population.

Variable	Lo	Med	*P*	Hi	*P*	*P* for trend	*P* for interaction
Sex							0.96
Female	Reference	0.58 (0.43,0.80)	<0.001	0.42 (0.29,0.61)	<0.0001	<0.0001	
Male	Reference	0.60 (0.37,0.99)	0.05	0.46 (0.28,0.76)	0.003	0.003	
Age group							0.38
20–44	Reference	0.63 (0.44,0.90)	0.01	0.56 (0.36,0.88)	0.01	0.86	
45–64	Reference	0.52 (0.37,0.74)	<0.001	0.32 (0.19,0.53)	<0.0001	<0.0001	
≥65	Reference	0.86 (0.42,1.76)	0.68	0.54 (0.25,1.15)	0.11	0.12	
Race							0.17
Other	Reference	0.74 (0.44,1.25)	0.26	0.68 (0.36,1.27)	0.22	0.19	
Black	Reference	1.07 (0.66,1.73)	0.79	0.80 (0.42,1.53)	0.49	0.65	
White	Reference	0.54 (0.37,0.77)	0.001	0.38 (0.27,0.52)	<0.0001	0.26	
Mexican	Reference	0.52 (0.23,1.19)	0.12	0.37 (0.11,1.34)	0.13	0.09	
Smoking							0.11
Never	Reference	1.01 (0.68,1.50)	0.95	0.43 (0.25,0.76)	0.004	0.002	
Former	Reference	0.80 (0.43,1.48)	0.47	0.61 (0.31,1.20)	0.15	0.16	
Current	Reference	0.52 (0.36,0.75)	<0.001	0.61 (0.38,0.96)	0.03	0.01	
BMI (kg/m^2^)							0.07
<25	Reference	0.38 (0.22,0.68)	0.001	0.31 (0.16,0.58)	<0.001	0.002	
25–30	Reference	0.58 (0.36,0.92)	0.02	0.34 (0.18,0.63)	<0.001	<0.001	
≥30	Reference	0.83 (0.56,1.23)	0.35	0.65 (0.44,0.96)	0.03	0.04	
Education level							0.43
High school diploma	Reference	0.83 (0.53,1.31)	0.42	0.64 (0.34,1.20)	0.16	0.13	
Below high school diploma	Reference	0.64 (0.41,0.99)	0.05	0.74 (0.40,1.36)	0.32	0.17	
Above high school diploma	Reference	0.59 (0.39,0.90)	0.01	0.41 (0.26,0.65)	<0.001	<0.001	

BMI, body mass index; CIAD, chronic inflammatory airway disease.

## 4 Discussion

For the first time, we utilized the NHANES database to delve into the relationship between CIAD and depressive symptoms and the role of dietary live microbe in the relationship. It was clarified that having CIAD increased the risk of developing depressive symptoms, while high levels of dietary live microbe reduced the risk of developing depressive symptoms in patients with CIAD. This moderating effect of dietary live microbe was most significant in the normal BMI group [OR 0.31, 95%CI (0.16, 0.58), *p* < 0.001].

The CIAD is a group of diseases characterized by non-specific chronic inflammation of the airways, including chronic bronchitis, emphysema, COPD, and bronchial asthma, with chronic cough, sputum, and chest tightness and wheezing as their main symptoms (23). A study utilizing the Charles Database in China university that the coexistence of multiple chronic diseases was positively associated with a positive risk of developing depressive symptoms, with an increased prevalence of about 45% (24). A meta-analysis demonstrated that diabetes increased the risk of depressive symptoms by about 25% (25). And in the study on impaired lung function, researchers found that people with impaired lung function had a 12.4% increased risk of depressive symptoms (26). Previous studies have shown that socioeconomic status is strongly associated with the development of depressive symptoms, with those of disadvantaged socioeconomic status having an increased risk of depressive symptoms in adulthood (27, 28). Educational level is also an important factor in mental health, with graduate students having more than six times the risk of anxiety and depression compared to the general population (29). In addition, smokers have more than twice the risk of developing depression symptoms at twice the risk of non-smokers (30). And in our study, the risk of depressive symptoms was 62% higher in patients with CIAD compared to those who did not have CIAD, and was especially below high school diploma and former smokers who had up to 2.85 and 2.5 times the risk of developing depressive symptoms. Compared to studies on other chronic comorbidities, patients with CIAD demonstrate a higher risk of developing depressive symptoms (25, 26). Specifically, CIAD patients with lower educational attainment or a history of smoking require heightened clinical vigilance for depressive symptom onset. Early psychological assessment should be prioritized in these subgroups to mitigate adverse outcomes.

**Figure 2:**
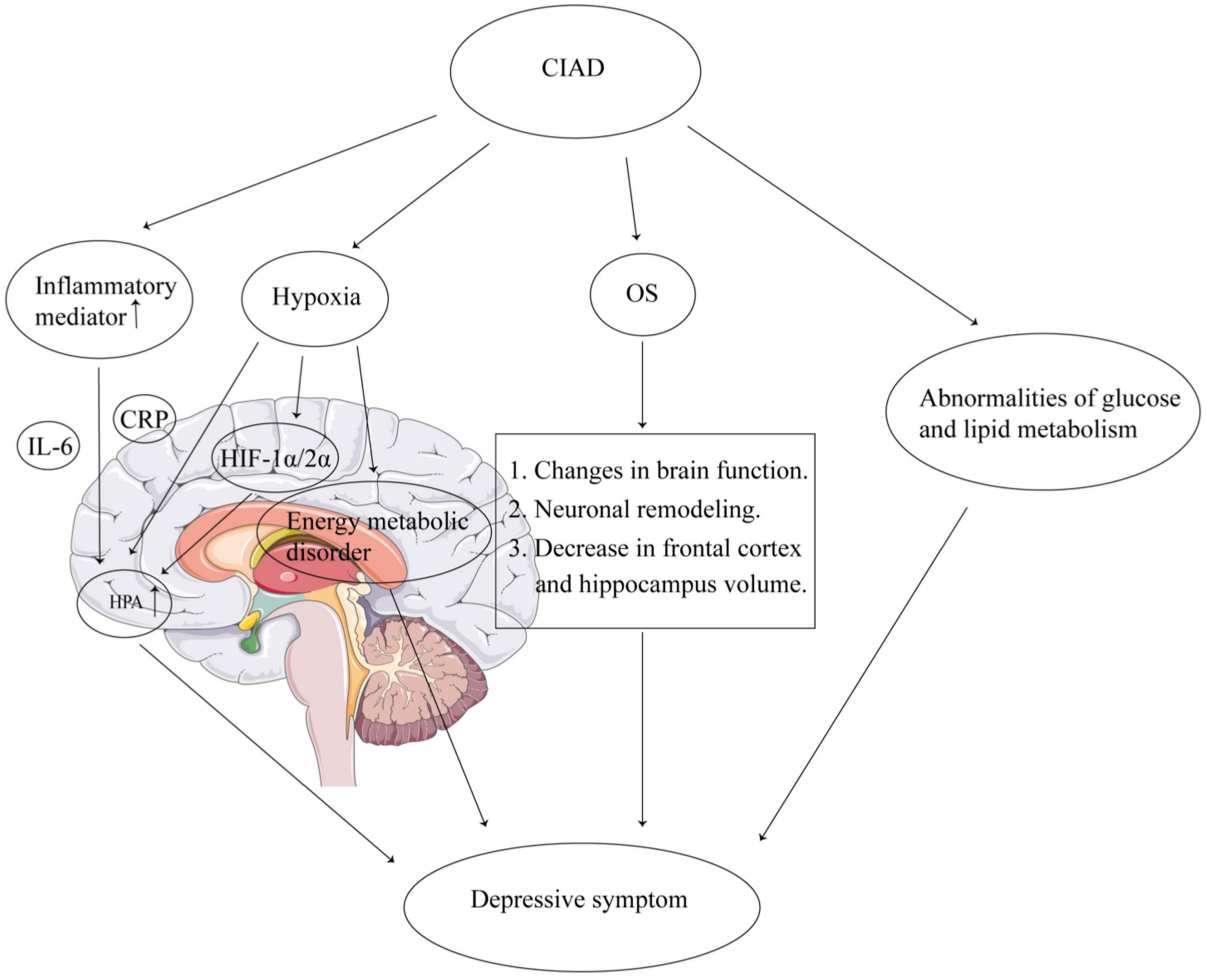
Mechanisms of the association between CIAD and depressive symptoms. CIAD, chronic inflammatory airway diseases. IL-6, interleukin-6. CRP, C-reactive protein. OS, oxidative stress. HPA, hypothalamic–pituitary–adrenal. HIF, hypoxia-inducible factor.

The pathophysiological mechanisms underlying the association between CIAD and depressive symptoms remain incompletely understood. Current evidence suggests the following potential pathways: First, CIAD patients exhibit upregulated systemic inflammation, particularly elevated interleukin-6 (IL-6) and C-reactive protein (CRP), which may activate the hypothalamic–pituitary–adrenal (HPA) axis, leading to excessive cortisol secretion. Chronic inflammation amplifies HPA axis hyperactivity, a hallmark of depression, by promoting glucocorticoid resistance and impairing negative feedback mechanisms (31–33). Second, hypoxia can activate the HPA axis directly or indirectly by increasing the expression of hypoxia-inducible factor (HIF) 1α/2α, which elevates cortisol levels *in vivo* increasing the risk of depressive symptoms (34, 35). In addition, hypoxia can disrupt brain energy metabolism as well as cause mitochondrial dysfunction in immune cells triggering depressive symptoms (36–38). Third, elevated levels of oxidative stress (OS) in CIAD, which can cause altered brain function, neuronal remodeling, and reduced frontal cortex and hippocampal volume, have been associated with the onset of depression (39). Furthermore, due to increased OS, activation of proinflammatory pathways also contributes to the development of depression (39). In addition, metabolic disorders (including disorders of glucose, lipid, and amino acid metabolism) are present in CIAD, and abnormalities in glucose and lipid metabolism are closely associated with depressive symptomatology (40–42) (Figure 2).

There may be a link between live microbe and depressive symptoms. Gut microbes may modulate brain states by influencing the release of glucagon-like peptide, cholecystokinin, adrenocorticotropin-releasing factor, neuropeptides, and other factors, and may play a role in ameliorating anxiety and depressive symptoms (43). A study published in Molecular Psychiatry by Xiaolong Wang’s team found significant changes in depression-like behavior and flora diversity in chronic ethanol model mice ingesting probiotics. By using adeno-associated virus to reduce hippocampal NLRP3 expression in mice and performing fecal microbiota transplantation with their gut microbiota, the researchers discovered that the gut microbiota of donor mice exposed to ethanol but not exhibiting depressive-like behaviors induced depressive-like behaviors, elevated peripheral inflammatory cytokines, and neuronal damage in recipient mice. This study reveals that gut flora can modulate ethanol exposure-induced depressive disorders through peripheral inflammation and hippocampal NLRP3 inflammatory vesicles. This study demonstrates the link between alcohol exposure-induced depression and changes in gut flora, which provides new therapeutic avenues for intervening in ethanol exposure-induced depression (44). Different gut microbe have variable effects on depressive symptoms. *Campylobacter jejuni* can produce c-FOS to cause anxiety and depression, whereas Lactobacillus and Bifidobacterium can significantly reduce depression (45, 46). In addition, previous studies have shown that antidepressant medications have a number of antimicrobial effects that can exert anxiolytic and depressive effects by affecting the gut microbiota (47, 48). Some antibiotics (e.g., quinolones, tetracyclines, etc.) can affect the development of anxiety and depressive symptoms, possibly due to the effect of antibiotics on the gut microbiota (49, 50).

Thus, there is a close correlation between live microbe, CIAD, and depressive symptoms, which may be related to the gut-lung axis and gut-brain axis. Studies have shown that disruption of the pulmonary epithelial barrier function in CIAD may lead to translocation of pathogenic bacteria, and translocated pathogens and pathogen-associated virulence factors may also enter the gastrointestinal tract to cause intestinal flora disruption (51). Moreover, intestinal microbial disruption is closely associated with the development of depressive symptoms (52). Our study found that high levels of dietary live microbe significantly negatively moderated the relationship between CIAD and depressive symptoms, with high levels of dietary live microbe reducing the risk of depressive symptoms by approximately 39% in patients with CIAD. Among the BMI group, high levels of dietary live microbe reduced the risk of CIAD by approximately 69% in CIAD patients with normal BMI, while patients with BMI ≥ 30 kg/m^2^ only reduced the risk by 35%. This may be related to the presence of disturbed gut microorganisms in obese patients, with a shift from the phylum Mycobacterium anomalum to the phylum Thick-walled Mycobacterium and thus an increased risk of depression (53). Furthermore, high levels of dietary live microbe reduced the risk of depressive symptoms in patients with CIAD, regardless of gender. Thus, the consumption of unpasteurized fermented foods or probiotic supplements in patients with CIAD is beneficial in reducing the risk of depressive symptoms, and the effect may be especially pronounced in people with normal BMI, 45–64 years of age, or with a high school education or higher.

This study has several strengths. First, the most significant strength of this study is that it contains a large sample that is nationally representative and can be generalized to various community populations. Second, this study adjusted for a variety of confounders such as age, sex, economic education level, common comorbidities, etc., and the findings remained significant, and subgroup analyses were performed to show that there was no interaction between subgroups for our findings. Finally, our study explored the relationship between dietary live microbe and depressive symptoms for the first time in patients with CIAD, and clearly demonstrated that a daily diet containing high levels of live microbe in patients with CIAD reduces the risk of depressive symptoms. This provides new insights and ideas for clinical prevention and treatment of depressive symptoms.

However, it is inevitable that this study has certain limitations. First, this study is a cross-sectional study and a causal relationship between the two cannot be deduced. Second, although many variables were included in this study for analysis, there may still be some variables that were not included causing some bias. Third, the dietary live microbe load in the study was based on participants’ assessment of the previous day’s food report, which may have recall bias and self-report bias. And it could not reflect the effects of changes in long-term dietary patterns, limiting the study of long-term health effects. Further research is needed to refine this component. Fourth, the NHANES database does not currently provide specific types of live microbe, so we are unable to explore the role of specific live microbe in the association between CIAD and depressive symptoms. Further research is needed to explore this area. Fifth, this study population did not include people under 20 years of age and over 65 years of age, which may lead to limitations in the applicability and generalizability of the findings to children, minors, and elderly populations. Future studies may need to balance these issues in their design to ensure that the findings more fully serve the public health needs of different age groups.

## 5 Conclusion

There is a strong relationship between dietary live microbe, CIAD, and depressive symptoms. Having CIAD can increase the risk of depressive symptoms, while high levels of dietary live microbe can reduce the risk of depressive symptoms in CIAD patients. Live microbe negatively modulate the effects of CIAD on depressive symptoms. This study provides new ideas for the prevention and treatment of depressive symptoms in patients with CIAD.

## Data Availability

The original contributions presented in the study are included in the article/Supplementary material, further inquiries can be directed to the corresponding author.

## Footnotes

^1^ https://wwwn.cdc.gov/nchs/nhanes/Default.aspx
